# Dysmenorrhea among working women and its effect on their work productivity and activity impairment

**DOI:** 10.1186/s42506-025-00199-7

**Published:** 2025-10-06

**Authors:** Hend Serya, Rania El-Kurdy, Ebrahim Serria, Abdel-Hady El-Gilany

**Affiliations:** 1 Industrial Medicine and Occupational Health, Public Health and Community Medicine Department, Faculty of Medicine, Mansoura University, Mansoura City, Egypt; 2 Woman’s Health and Midwifery Nursing Department, Faculty of Nursing, Mansoura University, Mansoura, Egypt; 3 Obstetrics and Gynecology Department, Damietta Faculty of Medicine, Al-Azhar University, Damietta, Egypt; 4 Public Health and Preventive Medicine, Public Health and Community Medicine Department, Faculty of Medicine, Mansoura University, Mansoura City, Egypt

**Keywords:** Dysmenorrhea, Working women, Absenteeism, Presenteeism, Work productivity, Daily activity impairment

## Abstract

**Background:**

Dysmenorrhea, or painful menstruation, is a common condition among women of reproductive age, significantly hindering their ability to work and participate in social, family, and sports activities. In Egypt, there is limited data on the frequency of dysmenorrhea among working women and its impact on their productivity. Therefore, this study aimed to investigate the prevalence of dysmenorrhea among working women, its determinants, and its effect on their work productivity and activity impairment.

**Methods:**

A cross-sectional study was conducted on 548 working women aged 20 to 45 years. Data were collected using an interview-based questionnaire that included sociodemographic, occupational, and clinical information, menstrual history, job stress assessment using the workplace stress scale, and work productivity evaluation with the work productivity and activity impairment questionnaire.

**Results:**

The dysmenorrhea rate was 66.1%, with 64.7% of those affected reporting moderate to severe pain. Key independent predictors of dysmenorrhea include young age (≤ 32 years), working overtime or extra jobs, experiencing workplace stress, having an early menarche (< 12 years), and having a family history of dysmenorrhea. Dysmenorrheic working women reported an absenteeism rate of 39.5%, a presenteeism rate of 96.1%, an overall work impairment rate of 96.4%, and a daily activity impairment rate of 94.2%. All of these rates were significantly higher in dysmenorrheic working women compared to those without the condition. Notably, the significant differences between the two groups increased with the severity of menstrual pain.

**Conclusion:**

Dysmenorrhea is a prevalent issue among working women in Egypt, adversely affecting their performance at work and in daily activities. Therefore, providing workplace rest areas equipped with pain relief options, implementing flexible work schedules or menstrual leave, and encouraging dysmenorrheic working women to seek medical care for severe pain that impairs their work or daily activities are highly recommended.

## Introduction

Globally, dysmenorrhea is a common gynecological condition affecting women of childbearing age [1]. It is frequently defined as colicky pain or cramping of uterine origin that occurs just before or during menstruation, usually localized in the lower abdomen, but it may also radiate to the lower back, legs, and inner thighs. This condition is often accompanied by additional symptoms, such as sweating, headaches, tachycardia, backache, mood swings, irritability, nausea, vomiting, and diarrhea [2, 3]. Dysmenorrhea can be classified based on its underlying cause: it is termed primary when both pelvic examination and ovulatory function are normal, or secondary when pelvic pathology is identified, such as adenomyosis, endometriosis, pelvic inflammatory disease, uterine fibroids, endometrial polyps, or cervical stenosis [4].

Between 30.0% and 90.0% of women report experiencing painful menstruation during their reproductive years, with very severe pain reported in 10.0% to 20.0% of cases, rendering them unable to perform their work or engage in family, social, and sports activities [1, 5]. Among working women, the rate of dysmenorrhea varies by country and occupation; 71.8% among Japanese workers [6], 80.7% among Chinese factory workers [7], 70.7% among Taiwanese hospital nurses [8], and 94.6% among working women in Upper Egypt [9]. The risk factors for dysmenorrhea are diverse and include young age, nulliparity, early onset of menarche (< 12 years), a family history of dysmenorrhea, prolonged or abnormal menstrual flow, smoking, lack of physical activity, moderate to high levels of stress, sleep disorders, as well as depression and anxiety [10, 11]. Furthermore, occupational factors such as high job strain, poor job security, and rotating or night shift work have also been associated with dysmenorrhea [7, 12].

Dysmenorrhea is associated with high levels of absenteeism, presenteeism, and limitations on daily activities. This results in significant economic losses due to expenses for medications and healthcare, as well as reduced productivity [9]. In a large cohort study involving 32,748 women aged 15–45, menstrual symptoms, particularly abdominal pain, were significantly associated with productivity loss due to absence from work (absenteeism) and productivity loss despite being present at work (presenteeism). Whereas the mean absenteeism accounted for 1.3 days of productivity loss per year, presenteeism contributed to 8.9 days of productivity loss [13]. Among the accommodations proposed for menstruating women are paid leave, flexible schedules, telework, affordable menstrual levels of absenteeism, presenteeism, and limitations on daily activities. This results in significant Korea, Spain, and Italy, have enacted menstrual leave laws [6].

Globally, women are becoming increasingly active in the workforce [14]. In Egypt, driven by economic pressures, the rate of women participating in the labor force has risen dramatically, reaching 15.6% of all workers by 2021 [15]. More than half of working women were engaged in professional, technical, and managerial positions or clerical occupations (52.0%), while 25.0% were employed in sales and services, 7.0% worked in skilled manual labor, and 8.0% were involved in agricultural activities [16].

Numerous studies in Egypt have examined the prevalence of dysmenorrhea among adolescent girls [17–19]. However, to our knowledge, few studies have focused on dysmenorrhea in working women [9, 20], and none have explored its impact on their work productivity. Studying the productivity of Egyptian working women is crucial, given their vital role in Egypt’s social and economic transformation. This study aimed to estimate the prevalence of dysmenorrhea among working women, identify the potential risk factors associated with it, and determine its effect on their work productivity and activity impairment.

## Methods

### Study design and settings

A cross-sectional study was conducted from June to August 2024 at two university hospitals: Mansoura University Hospital and Al-Azhar University Hospital—New Damietta. These hospitals are located in the Nile Delta region of Egypt and provide healthcare services to residents of Dakahlia and Damietta Governorates, as well as to those in nearby governorates.

### Sample size

A sample size of 548 workers was calculated using MedCalc software, version 14.8.1. This calculation was based on a 94.6% prevalence of dysmenorrhea among Egyptian working women [9] with an alpha error of 5.0%, beta error of 20.0%, 5.0% precision, a design effect of 2.0, and a 10.0% non-response rate.

### Recruitment and eligibility criteria

Working women were recruited and interviewed while attending the outpatient clinic in the Obstetrics and Gynecology departments, including those seeking consultations for various reasons such as dysmenorrhea management, premarital screening, gynecological health check-ups, ovulation monitoring, or exploring family planning options. Additionally, patients’ relatives and female hospital staff members (e.g., physicians, nurses, pharmacists, technicians, or service workers) who were at the hospital during the study and met the eligibility criteria were also included in this research. Eligible participants were Egyptian working women aged 18 to 45 with at least one year of work experience, who had menstruated within the past three months and agreed to participate in the study. Conversely, working women who were using hormonal pills or intrauterine devices as contraceptives, experiencing abnormal uterine bleeding, undergoing fertility treatment, on sick leave, exhibiting perimenopausal symptoms, had undergone a hysterectomy or ovariectomy, or were pregnant or breastfeeding, were excluded from the study.

### Study tools

Data were collected using an interview-based questionnaire, which consisted of four sections as follows:

- The first section included sociodemographic, occupational, and clinical data such as age, residence, educational level, marital status, parity, smoking history, current occupation, type of workplace (public, private, or self/family), working duration (years), weekly working hours, shift work (day, night, or rotating), working overtime or extra job, and physician-diagnosed health problems emphasizing the gynecological ones.
  In the second section, the self-reported menstrual history of the study participants was recorded including age at menarche, family history of dysmenorrhea, dysmenorrhea over the past three months, concurrent clinical symptoms, its severity using the verbal multidimensional scoring system (VMS), pain relief methods only in the participants declaring dysmenorrhea, as well as whether they had to take sick leave for their symptoms. The VMS scoring system ranged from grades 0 to 3 and considered the effect of pain on women’s daily activity, systemic symptoms, and whether analgesia was required. Grade 0 indicates the absence of dysmenorrhea, while grades 1, 2, and 3 correspond to mild, moderate, and severe dysmenorrhea, respectively. Dysmenorrhea of grade 1, or mild dysmenorrhea, is characterized by painful menstruation that rarely requires analgesics and hardly affects women's daily activities. The second grade is described as moderate menstrual pain that disrupts women's daily activities, but that is effectively relieved by analgesics. Menstrual pain of Grade 3, also known as severe dysmenorrhea, refers to extremely painful menstruation accompanied by vegetative symptoms (e.g., headache, nausea, asthenia, diarrhea, vomiting), inhibiting daily activities, and not relieved by analgesics [21].
- The third section of the questionnaire pertained to the workplace stress scale (WSS), developed by the Marlin company and the American Institute of Stress. It was used to outline job stress levels among working women. WSS is an 8-question Likert-type scale with 5 responses to each question. Each response option is scored from (1–5), i.e., (1) never, (2) rarely, (3) sometimes, (4) often, and (5) very often. Workplace stress was categorized based on the calculated score as follows: Yes (scores ranging from 16 to 40) and No (scores of 15 or lower) [22]. A validated Arabic version of the scale was used, adapted from an earlier Egyptian study, where reliability was 0.80 for the entire scale [23].
- In the fourth section, the work productivity and activity impairment questionnaire (WPAI) was used to measure working women's impairments in work and daily activities during the last menstrual period [24, 25]. The questionnaire incorporated six questions that elicited the following: (Q1) employment status; (Q2) hours missed from work during the last menstrual period; (Q3) hours missed from work due to reasons other than the menstrual period; (Q4) total hours worked; and two questions that assessed the extent to which the menstrual period affected productivity at work and daily activities, rated on a scale from 0 to 10; (Q5) measured the degree to which the menstrual period affected productivity while working, and (Q6) inquired about the degree to which the menstrual period limited the performance of regular daily activities involving exercising, shopping, childcare, studying, or working around the house. We modified the questionnaire by omitting the first question, as all the participants in our study sample were working. The WPAI yielded four outcomes as follows: (1) Absenteeism (work time missed) = Q2/(Q2 + Q4); (2) Presenteeism (impairment at work/reduced on-the-job effectiveness) = Q5/10; (3) Overall work impairment (absenteeism + presenteeism) = Q2/(Q2 + Q4) + [(1—(Q2/(Q2 + Q4)) × (Q5/10)]; (4) daily activity impairment = Q6/10. The scores were multiplied by 100 to convert them into percentages, with a higher percentage representing greater impairment and less productivity, i.e., worse outcomes (0% = no impairment to 100% = total impairment).
- After completing the interview, anthropometric measurements were collected, including height and weight. Body weight was measured to the nearest 0.5 kg, with participants wearing light clothing and no shoes, using a digital weighing scale. Height was also measured without shoes, using a steel measuring tape to the nearest 0.5 cm from the participant’s head to toe in an upright standing position. The Body Mass Index (BMI) is calculated by dividing weight in kilograms by the square of height in meters (kg/m^2^). The BMI for normal weight ranges from 18.5 to 24.9 kg/m^2^, 25.0 to 29.9 kg/m^2^ for overweight, and 30 kg/m^2^ and above for obesity [26].

### Statistical analysis

Data analysis was performed using IBM SPSS Statistics for Windows, version 28 (IBM Corp., Armonk, N.Y., USA). Categorical data were represented as numbers and percentages of the total, while quantitative data were tested for normality using the Kolmogorov–Smirnov test and were reported as mean ± standard deviation or median (minimum–maximum) based on the data distribution. The Chi-square and Monte Carlo tests were employed to examine the relationships between qualitative variables as needed. All significant variables identified in the bivariate analysis were incorporated into a multivariate logistic regression model using the forward Wald method to identify key independent predictors of dysmenorrhea. Crude and adjusted odds ratios and their 95% confidence intervals were calculated. The Mann–Whitney and Kruskal–Wallis tests were utilized for quantitative variables to compare scores across WPAI domains. The correlation between scores of WPAI domains and background characteristics of dysmenorrheic working women was tested using Pearson’s correlation. Categorical variables were entered as dummy variables with codes as: age (≤ 32 years = 0, > 32 years = 1), educational level (≤ secondary = 0, > secondary = 1), marital status (married = 0, unmarried = 1), parity(≥ 1 = 0, nullipara = 1), shift work (rotating shift = 0, day shift = 1), working overtime or extra jobs (yes = 0, no = 1), workplace stress (no = 0, 1 = yes), gynecological morbidities (no = 0, 1 = yes). Significant correlations were included in a multivariate stepwise linear regression analysis to identify independent predictors of WPAI domains among working women with dysmenorrhea. A p-value of ≤ 0.05 was deemed significant for all statistical tests.

## Results

The participants' mean age was 31.8 ± 6.6 years, ranging from 20.0 to 45.0 years, and their residences in urban or rural areas were almost equal. More than half were educated to secondary school or less (61.3%), married (66.4%), and primiparous/multiparous (66.6%). All participants were non-smokers. Among the recruited participants, over two-fifths were hospital staff members (42.3%), and more than half worked in governmental workplaces (61.9%). Nearly half of the participants held medium-skilled jobs (49.3%) with a median working duration of 7 years. The majority of respondents worked 48 h or fewer per week (67.2%) and were engaged in day shifts (69.5%), while only 19.2% worked overtime or held extra jobs. The body mass index of the working women ranged from 20.3 to 44.4 kg/m^2^, with a mean of 28.3 ± 3.5. Among the participants, 5.3% reported a physician's diagnosis of gynecological health problems (Table 1).

**Table 1:** Background characteristics of the studied working women, Al-Azhar University Hospital- New Damietta and Mansoura University Hospital, Mansoura, Egypt, 2024

Characteristic	Total number = 548
	No. (%)
**Age (years)**	
Range	20- 45
Mean ± SD	31.8 ± 6.6
**Residence**	
Rural	271 (49.5)
Urban	277 (50.5)
**Educational level**^**a**^	
≤ secondary	336 (61.3)
> secondary	212 (38.7)
**Marital status**	
Married	364 (66.4)
Unmarried^b^	184 (33.6)
**Parity**	
≥ 1	365 (66.6)
Nullipara	183 (33.4)
**Smoking**	
Non-smokers	548 (100.0)
**Recruited participants**	
Hospital staff	232 (42.3)
Patients’ relatives	178 (32.5)
From the gynecologic clinic	138 (25.2)
**Type of workplace**	
Governmental	339 (61.9)
Private	141 (25.7)
Self/family	68 (12.4)
**Type of job**^**c**^	
Low skill	10 (1.8)
Medium skill	270 (49.3)
High skill	268 (48.9)
**Working duration (years)**, Median (Min–Max)	7 (1- 25)
**Weekly working hours**^**d**^	
≤ 48 h	368 (67.2)
> 48 h	180 (32.8)
**Shift work**	
Day shifts	381 (69.5)
Rotating shifts	167 (30.5)
**Working overtime or extra jobs**	105 (19.2)
**BMI**^**e**^** (kg/m**^**2**^**)**	
Range	20.3- 44.4
Mean ± SD	28.3 ± 3.5
** Gynecological morbidities**^**f**^	29 (5.3)

^a^The category of ≤ secondary education involves illiterate women (*n* = 3), can read and write (*n* = 5), have completed primary education (*n* = 7), preparatory education (*n* = 8), and secondary education (*n* = 135). The category of > secondary education includes those who have attended intermediate institutes (*n* = 178) and those who have completed university education or higher (*n* = 212)

^b^Unmarried includes single, widowed, and divorced

^c^According to the International Standard Classification of Occupations (ISCO*-*88)

^d^According to Egyptian labor law, the maximum working hours are 8 hours per day or 48 hours per week for a six-day workweek

^e^BMI Body mass index

^f^Polycystic ovary syndrome (*n* = 8); fibroid (*n* = 6); pelvic inflammatory disease (*n* = 6); ovarian cyst (*n* = 4); endometriosis (*n* = 3); and cystocele (*n* = 2)

The dysmenorrhea rate was 66.1% among working women in the past 3 months, with moderate severity being more common (38.7%), followed by mild (35.3%) and severe dysmenorrhea (26.0%). Backache was the most frequent symptom associated with dysmenorrhea (64.6%), followed by headache (47.0%), fatigue (27.9%), nervousness (26.5%), nausea/vomiting (26.2%), depressed mood (22.7%), and breast tenderness/swelling (18.8%). Drinking hot liquids/herbal fluids (90.3%) and taking medications (analgesics and/or antispasmodics) (63.0%) ranked as the most widely used pain relief methods in dysmenorrheic working women followed by heat pack application (38.4%) and rest (22.1%), while taking hot shower (8.0%), exercising (8.0%) and messaging (lower abdomen and/or back) (5.2%) were tried by few women. Nearly one-fifth of working women complaining of dysmenorrhea and/or its concurrent symptoms reported taking sick leaves (21.3%). The mean menarche age of working women was 12.2 ± 1.6, with 52.6% reporting a family history of dysmenorrhea (Table 2).

**Table 2:** Menstrual profile of the studied working women, Al-Azhar University. Hospital- New Damietta and Mansoura University Hospital, Egypt, 2024

Variable	Total number = 548
	No. (%)
Dysmenorrhea^a^	362 (66.1)
Menarche age, mean ± SD	12.2 ± 1.6
Family history of dysmenorrhea	288 (52.6)
	Dysmenorrheic women = 362
	No. (%)
Dysmenorrhea severity	
Mild	128 (35.3)
Moderate	140 (38.7)
Severe	94 (26.0)
Dysmenorrhea concurrent symptoms^b^	
Backache	234 (64.6)
Headache	170 (47.0)
Fatigue	101 (27.9)
Nervousness	96 (26.5)
Nausea/vomiting	95 (26.2)
Depressed mood	82 (22.7)
Breast tenderness/swelling	68 (18.8)
Appetite changes	48 (13.3)
Acne	47 (13.0)
Dizziness	43 (11.9)
Insomnia	34 (9.4)
Artharlagia	31 (8.6)
Abdominal bloating	26 (7.2)
Diarrhea/constipation	22 (6.1)
Dysuria/polyuria	11 (3.0)
Loss of concentration	10 (2.8)
Fainting	4 (1.1)
Pain relief methods^b^	
Hot liquids/herbal fluids	327 (90.3)
Medications (analgesics and/or antispasmodics)	228 (63.0)
Heat pack application	139 (38.4)
Rest	80 (22.1)
Hot shower	29 (8.0)
Exercise	29 (8.0)
Message (lower abdomen/back)	19 (5.2)
Sick leave^a^	77 (21.3)

^a^In the past three months

^b^Categories are not mutually exclusive

In the bivariate analysis, dysmenorrhea was found to be significantly more common among working women who were 32 years of age or younger, unmarried, nulliparous, had worked for 7 years or less, worked more than 48 h per week, engaged in rotating shifts, worked overtime or extra jobs, experienced workplace stress, had an early menarche (< 12 years), and had a family history of dysmenorrhea. These significant risk factors were entered into a multivariate stepwise logistic regression analysis which revealed that young age (≤ 32 years) (AOR 2.2, 95% CI: 1.5- 3.3), working overtime or extra jobs (AOR 2.6, 95% CI: 1.4- 4.9), experiencing workplace stress (AOR 2.8, 95% CI: 1.8- 4.3), having early menarche (< 12 years) (AOR 2.8, 95% CI: 1.8- 4.5), and having a family history of dysmenorrhea (AOR 2.3, 95% CI: 1.5–3.4) were all independent risk factors for the occurrence of dysmenorrhea among working women (Table 3).

**Table 3:** Bivariate and multivariate analysis of factors associated with dysmenorrhea among working women, Al-Azhar University Hospital- New Damietta and Mansoura University Hospital, Egypt, 2024

Variable	Total	Dysmenorrhea	*Bivariate Analysis*		*Multivariate Analysis*	
		No. (%) ^a^	*p-value*	*COR (95%CI)*	*p-value*	*AOR (95%CI)*
Overall	548	362 (66.1)				
Age (years)						
≤ 32	304	226 (74.3)	< 0.001^**^	2.3 (1.6- 3.3)	< 0.001^**^	2.2 (1.5- 3.3)
> 32 (r)	244	136 (55.7)				
Residence						
Rural	271	178 (65.7)	0.854	0.9 (0.7- 1.4)		
Urban (r)	277	184 (66.4)				
Educational level						
> secondary	212	150 (70.8)	0.065	1.4 (0.9- 2.0)		
≤ secondary (r)	363	212 (63.1)				
Marital status						
Unmarried	184	136 (73.9)	0.006^*^	1.7 (1.2- 2.6)		
Married (r)	364	226 (62.1)				
Parity						
Nullipara	183	142 (77.6)	< 0.001^**^	2.3 (1.5- 3.4)		
≥ 1 (r)	365	220 (60.3)				
Type of workplace						
Governmental	339	214 (63.1)	0.986	1.0 (0.6- 1.7)		
Private	141	105 (74.5)	0.094	1.7 (0.9- 3.2)		
Self/family (r)	68	43 (63.2)				
Type of job						
Low skill	10	6 (60.0)	0.636	0.7 (0.2- 2.7)		
Medium skill	270	176 (65.2)	0.627	0.9 (0.6- 1.3)		
High skill (r)	268	180 (67.2)				
Working duration (years)						
≤ 7	285	212 (74.4)	< 0.001^**^	2.2 (1.5- 3.1)		
> 7 (r)	263	150 (57.0)				
Weekly working hours						
> 48 h	180	134 (74.4)	0.004^*^	1.8 (1.2- 2.7)		
≤ 48 h (r)	368	228 (62.0)				
Shift work						
Rotating shifts	167	121 (72.5)	0.036^*^	1.5 (1.1- 2.8)		
Day shifts (r)	381	241 (63.3)				
Working overtime or extra jobs						
Yes	105	90 (85.7)	< 0.001^**^	3.7 (2.1- 6.7)	0.002^*^	2.6 (1.4- 4.9)
No (r)	443	272 (61.4)				
Workplace stress						
Yes	396	291 (73.5)	< 0.001^**^	3.2 (2.1- 4.7)	< 0.001^**^	2.8 (1.8- 4.3)
No (r)	152	71 (46.7)				
BMI categories						
Obese	184	122 (66.3)	0.168	1.4 (0.9- 2.4)		
Overweight	273	188 (68.9)	0.053	1.6 (1.0- 2.6)		
Normal weight (r)	90	52 (57.8)				
Gynecological morbidities						
Yes	29	22 (75.9)	0.252	1.6 (0.7- 3.8)		
No (r)	519	340 (65.5)				
Menarche age (years)						
< 12	185	148 (80.0)	< 0.001^**^	2.8 (1.8- 4.2)	< 0.001^**^	2.8 (1.8- 4.5)
12–14 (r)	317	187 (59.0)				
> 14	46	27 (58.7)	0.969	0.9 (0.5–1.8)		
Family history of dysmenorrhea						
Yes	288	221 (76.7)	< 0.001^**^	2.8 (1.9- 4.0)	< 0.001^**^	2.3 (1.5- 3.4)
No (r)	260	141 (54.2)				

*BMI* Body mass index, *r* Reference category, *COR* Crude odds ratio, *AOR* Adjusted odds ratio

^a^Percentages were calculated using row totals

^*^p ≤ 0.05 is statistically significant

^**^p < 0.001 is highly statistically significant

Dysmenorrheic working women reported an absenteeism rate of 39.5%, a presenteeism rate of 96.1%, an overall work impairment rate of 96.4%, and a daily activity impairment rate of 94.2%. All of these rates were significantly higher in dysmenorrheic working women compared to those without the condition, and these rates also significantly increased with increasing menstrual pain severity (*p* < 0.001). Moreover, dysmenorrheic working women exhibited significantly higher median scores across all domains of WPAI compared to their counterparts without dysmenorrhea. These scores significantly increased with the severity of menstrual pain (*p* < 0.001), indicating reduced work productivity and greater impairment in daily activities among dysmenorrheic working women. Additionally, the median weekly absenteeism hours were significantly higher among dysmenorrheic working women than in those without dysmenorrhea (Table 4).

**Table 4:** Work productivity and activity impairment among working women during their last menstrual period

WPAI domain	Dysmenorrhea		*p-value*	Dysmenorrhea severity, n = 362			*p-value*
	Yes *n *= 362	No *n* = 186		Mild *n* = 128	Moderate *n* = 140	Severe *n* = 94	
	No. (%)	No. (%)		No. (%)	No. (%)	No. (%)	
Absenteeism	143 (39.5)	24 (12.9)	< 0.001^**^	5 (3.9)	54 (38.6)	84 (89.4)	< 0.001^**^
Presenteeism	348 (96.1)	104 (55.9)	< 0.001^**^	115 (89.8)	140 (100.0)	93 (98.9)	< 0.001^**^
Overall work impairment	349 (96.4)	110 (59.1)	< 0.001^**^	115 (89.8)	140 (100.0)	94 (100.0)	< 0.001^**^
Daily Activity Impairment	341 (94.2)	100 (53.8)	< 0.001^**^	108 (84.4)	140 (100.0)	94 (100.0)	< 0.001^**^
WPAI domain score	Median (Min–Max)		*p-value*	Median (Min–Max)			*p-value*
Percent absenteeism	0.0 (0.0–50.0)	0.0 (0.0–38.9)	< 0.001^**^	0.0 (0.0–16.7) ^ab^	0.0 (0.0–33.0)^ac^	20.0 (0.0–50.0)^bc^	< 0.001^**^
Percent presenteeism	40.0 (0.0- 100.0)	10.0 (0.0- 40.0)	< 0.001^**^	20.0 (0.0–50.0)^ab^	50.0 (10.0–70.0)^ac^	80.0 (0.0–100.0)^bc^	< 0.001^**^
Percent overall work impairment	50.0 (0.0–100.0)	10.0 (0.0–57.0)	< 0.001^**^	20.0 (0.0–57.0)^ab^	50.0 (10.0–70.0)^ac^	83.0(25.0–100.0)^bc^	< 0.001^**^
Percent daily activity Impairment	40.0 (0.0–100.0)	10.0 (0.0–50.0)	< 0.001^**^	10.0 (0.0–60.0)^ab^	40.0(10.0–70.0)^ac^	80.0 (0.0–100.0)^bc^	< 0.001^**^
Abseentesim hours/week	0.0 (0.0–30.0)	0.0 (0.0–14.0)	< 0.001^**^	0.0 (0.0–7.0)^ab^	0.0 (0.0–16.0)^ac^	9.0 (0.0–30.0)^bc^	< 0.001^**^

Superscript letters (a, b, and c) indicate significant differences between the corresponding groups within the same row, as determined by the Mann–Whitney test

*WPAI* Work Productivity and Activity Impairment

^**^p < 0.001 is highly statistically significant

Absenteeism was significantly correlated with shift work, working overtime or extra jobs, and the presence of gynecological morbidities. In addition, age, educational level, marital status, parity, workplace stress, and gynecological morbidities were significantly correlated with presenteeism. Furthermore, overall work impairment showed a significant positive correlation with educational level, marital status, parity, workplace stress, and gynecological morbidities. Daily activity impairment also showed a significant positive correlation with parity, workplace stress, and gynecological morbidities (Table 5).

**Table 5:** Correlation between scores of WPAI domains and background characteristics of dysmenorrheic working women

Characteristic	Absenteeism^a^		Presenteeism^a^		Overall work impairment^a^		Daily activity impairment^a^	
	*n* = 143		*n* = 348		*n* = 349		*n* = 341	
	*r*	*p-value*	*r*	*p-value*	*r*	*p-value*	*r*	*p-value*
Age (years)	0.12	0.138	- 0.11	0.048^*^	- 0.10	0.061	- 0.08	0.120
Educational level	- 0.02	0.770	0.11	0.035^*^	0.12	0.027^*^	0.10	0.065
Marital status	- 0.03	0.725	0.13	0.014^*^	0.13	0.018^*^	0.08	0.140
Parity	- 0.01	0.929	0.18	< 0.001^**^	0.17	< 0.001^**^	0.15	0.006^*^
Shift work	0.18	0.031^*^	0.03	0.544	0.03	0.579	0.02	0.683
Working overtime or extra jobs	0.33	< 0.001^**^	0.05	0.361	0.05	0.339	0.06	0.234
Workplace stress	- 0.08	0.357	0.23	< 0.001^**^	0.22	< 0.001^**^	0.22	< 0.001^**^
Gynecological morbidities	0.19	0.020^*^	0.24	< 0.001^**^	0.23	< 0.001^**^	0.25	< 0.001^**^

*WPAI*: Work Productivity and Activity Impairment

^a^log transformed score

^*^p ≤ 0.05 is statistically significant

^**^p < 0.001 is highly statistically significant

Working overtime or extra jobs and gynecological morbidities were significant independent predictors of absenteeism. In contrast, factors such as parity, workplace stress, and gynecological morbidities significantly predicted other domains of WPAI, including presenteeism, overall work impairment, and daily activity impairment (Table 6).

**Table 6:** Linear stepwise regression of significant independent predictors of WPAI domains among dysmenorrheic working women

Variable	Absenteeism^a^		Presenteeism^a^		Overall work impairment^a^		Daily activity impairment^a^	
	*n* = 143		*n* = 348		*n* = 349		*n* = 341	
	*B* (95% CI)	*p-value*	*B* (95% CI)	*p-value*	*B* (95% CI)	*p-value*	*B* (95% CI)	*p-value*
Parity	-	-	0.24 (0.11–0.38)	< 0.001^**^	0.24 (0.11–0.38)	< 0.001^**^	0.20 (0.06–0.34)	0.006^*^
Working overtime or extra jobs	0.34 (0.17–0.50)	< 0.001^**^	-	-	-	-	-	-
Workplace stress	-		0.47 (0.30–0.64)	< 0.001^**^	0.48 (0.30–0.65)	< 0.001^**^	0.46 (0.28–0.64)	< 0.001^**^
Gynecological morbidities	0.26 (0.04- 0.48)	0.020^*^	0.54 (0.26–0.81)	< 0.001^**^	0.50 (0.22- 0.78)	< 0.001^**^	0.56 (0.28–85)	< 0.001^**^
Constant	2.59		3.15		3.18		3.13	
Significance	F = 11.20, *p* < 0.001		F = 18.23, *p* < 0.001		F = 17.00, *p* < 0.001		F = 15.61, *p* < 0.001	
R^2^	0.138		0.137		0.129		0.122	

*WPAI*: Work Productivity and Activity Impairment

^a^log-transformed score

^*^p ≤ 0.05 is statistically significant

^**^p < 0.001 is highly statistically significant

## Discussion

Dysmenorrhea is a prevalent problem that affects a significant percentage of women during their reproductive age, profoundly impacting their lives and health [1]. Nearly two-thirds of the working women in this study reported experiencing dysmenorrhea in the past three months (66.1%). Epidemiological studies conducted across various countries and occupations have reported a wide range of dysmenorrhea rates, varying from 14.5% to 94.6% [1, 5–9, 27–29]. Several factors may explain these discrepancies, including demographic variations, geographical location, and the cultural background of the studied population. Additionally, differences in data collection methods, the recall period for dysmenorrhea occurrence, and individual pain perception levels may contribute to these inconsistencies. As for the severity of dysmenorrhea, 64.7% of working women experiencing this condition reported their pain as moderate to severe. This figure was lower than the 86.0% reported in an earlier study conducted in Turkey [28], but higher than the rates observed in Japan (18.3%) [30], Spain (25.9%) [1], and Australia (44.2%) [31]. The variation in pain intensity across these studies likely reflects differences in how participants perceive pain, as well as the different pain intensity scales used to measure the severity of pain [32].

In the current study, working women reported experiencing a range of bothersome symptoms associated with dysmenorrhea, including backache (64.6%), headache (47.0%), fatigue (27.9%), nervousness (26.5%), nausea/vomiting (26.2%), depressed mood (22.7%), and breast tenderness/swelling (18.8%). Previous studies corroborated our findings, indicating that similar symptoms are prevalent among working women from diverse cultural backgrounds [6, 33]. Drinking hot liquids/herbal fluids (90.3%) and taking medications (analgesics and/or antispasmodics) (63.0%) were the most widely used methods for alleviating menstrual pain among dysmenorrheic working women, followed by applying heat packs (38.4%) and resting (22.1%). These data corroborated other studies conducted in Egypt [9] and France [29]. However, this contradicted the findings of Wuni et al., who revealed that only 20.8% of nurse and midwife trainees in Ghana utilize pharmacological methods, such as nonsteroidal anti-inflammatory drugs and/or antispasmodics, whereas 13.4% use nonpharmacological methods, the most commonly used being sleep (59.4%) and applying hot objects or liquids to the abdomen (50.0%) [32].

Our study, consistent with previous research [8, 27, 28], demonstrated that young working women (aged 32 years or younger) have higher odds of experiencing dysmenorrhea, with an AOR of 2.2. This observation may be attributed to the fact that, as women age, both the frequency of dysmenorrhea episodes and the use of coping strategies tend to increase. Consequently, women experiencing dysmenorrhea can be viewed as specialists with embodied knowledge, where their personal experiences shape their subjective perception and tolerance of dysmenorrhea, ultimately influencing its prevalence [8].

Our results revealed that early menarche (< 12 years) was an independent risk factor for dysmenorrhea among working women, with an AOR of 2.8. This finding was in coherence with previous observations [8, 34]. In contrast, Yöndem & Çıtak Bilgin in Turkey found that nurses suffering from dysmenorrhea had a higher mean age of menarche compared to those who did not. They attributed this variation in the menarche age to several factors, including hereditary traits, socioeconomic conditions, environmental influences, and overall health status [28]. Having a family history of dysmenorrhea was another independent predictor of dysmenorrhea in our study, with an AOR of 2.3. A similar result was also reported in Turkey [28] and Ghana [32].

As evidenced by previous studies [7, 30], our research findings indicated that exposure to workplace stress significantly increased the likelihood of dysmenorrhea among working women. Furthermore, the multivariate linear regression analysis demonstrated that it was an independent predictor of WPAI domains, including presenteeism, overall work impairment, and daily activity impairment. This observation may be explained by the fact that during periods of stress, the body produces excessive adrenal hormones, estrogen, progesterone, and prostaglandins, causing uterine muscle tension and excessive uterine contractions, resulting in pain [35].

Nearly one-fifth of working women in the present study reported taking sick leave in the past three months due to dysmenorrhea and/or its associated symptoms (21.3%). Sick leave rates varied across different studies, with women in Upper Egypt reporting a rate of 43.0% [9] and Turkey reporting a rate of 9.7% [28]. Furthermore, the rate of reported absenteeism among dysmenorrheic working women during their last menstrual period in our sample was nearly 40.0%. A lower rate was observed in the Netherlands (13.8%) [13], while a higher rate was found among US employees (45.2%) [33]. This variation in sick leave and absenteeism rates across studies may be attributed to differences in methodologies and tools used to estimate these rates, the recall periods for sick leave and absenteeism, as well as variations in working conditions, job roles, and working hours across various occupations, both within the same country and between different countries.

In this study, 96.1% of working women with dysmenorrhea reported experiencing presenteeism during their last menstrual period. This finding closely mirrored the research conducted by Schoep et al., who revealed that 80.7% of women in the Netherlands experienced presenteeism and decreased productivity during their menstrual period [13]. In contrast, de Arruda et al. in Brazil reported a lower rate of 44.2% [36]. This relatively high rate of presenteeism observed in our study may be attributed to several factors. First, the challenges of discussing a gender-specific condition, especially with male employers, may lead to prioritizing privacy over well-being. Second, these women may fear job loss, which can put them at a disadvantage during hiring and career advancement. Finally, their absence from work may have negative financial implications for both the women and their employers.

Our study revealed a significant negative correlation between presenteeism and age, a finding supported by Schoep et al. [13]. This result may be attributed to the shorter tenure of younger workers, their desire to establish trust and credibility with employers, and the perception that taking sick leave might be a sign of underperformance and inefficiency.

Dysmenorrheic working women in the current study demonstrated significantly lower work productivity compared to their counterparts without dysmenorrhea. Furthermore, productivity declined as the severity of menstrual pain increased. These findings were in line with an Australian study that declared women with dysmenorrhea were 50.0% more likely to experience reduced work performance and twice as likely to report higher absenteeism than their peers [31]. In the same context, de Arruda et al. in Brazil stated that women with higher menstrual pain intensity were more likely to present with presenteeism (OR = 1.29) [36]. Additionally, working women with dysmenorrhea experienced a significantly higher rate of daily activity impairment compared to those without the condition (94.2% vs. 53.8%). Studies conducted in Spain [1], France [29], and Brazil [37] corroborated this finding.

In multivariate linear regression analysis, the present study showed that gynecological morbidities were a significant independent predictor of WPAI domains. Similarly, Lozano-Lozano et al. [38] and Soliman et al.[39] reported that women with endometriosis experience significant impairments in productivity at work and daily activities compared to those without the condition. In the same context, Hasselrot et al. in Sweden indicated that patients with uterine fibroids were significantly affected professionally and privately during menstruation, missing a higher percentage of work time each menstrual cycle, and showing greater impairment in work-related activities compared to controls [40].

### Strengths and limitations

To the best of our knowledge, this is the first study conducted in Egypt to examine the relationship between dysmenorrhea and working productivity among working women. However, this study had some limitations. First, the inherent weakness of the cross-sectional design is that it does not allow for causal inferences. Second, lifestyle factors such as diet and physical exercise were not investigated. Third, most variables in this study were measured based on participants’ self-reports, which may have introduced recall bias. Finally, as we interviewed women during hospital visits, the use of convenience sampling may have led to selection bias. Therefore, future research should employ a nationally representative random sampling technique.

## Conclusion and recommendations

Nearly seven out of ten working women in the present study suffered from dysmenorrhea in the past three months, with more than three-fifths reporting moderate to severe pain. Factors that significantly increase the likelihood of suffering from dysmenorrhea include being younger than 32 years, working overtime or extra jobs, experiencing workplace stress, having an early onset of menstruation (before the age of 12), and having a family history of dysmenorrhea. Dysmenorrheic working women exhibited significantly lower work productivity and more impairment in daily activities compared to those without the condition. These findings underscore the urgent need for appropriate measures, including the provision of rest areas in the workplace equipped with pain relief options such as heat pads, hot beverages, shower facilities, and medications; implementing flexible work schedules or menstrual leave, allowing workers to manage their workloads around their menstrual cycles; encouraging dysmenorrheic working women to seek medical care when experiencing severe pain that significantly impairs their work or daily activities; and incorporating menstrual self-care into occupational safety and health training.

## Data Availability

The datasets used and analyzed during this study are available from the corresponding author upon reasonable request.

## Abbreviations

AOR: Adjusted odds ratio
BMI: Body mass index
CI: Confidence interval
VMS: Verbal multidimensional scoring
WPAI: Work productivity and activity impairment
WSS: Workplace stress scale
